# With or without Feedback?—How the Presence of Feedback Affects Processing in Children with Developmental Language Disorder

**DOI:** 10.3390/brainsci13091263

**Published:** 2023-08-30

**Authors:** Lauren S. Baron, Asiya Gul, Yael Arbel

**Affiliations:** MGH Institute of Health Professions, Boston, MA 02129, USA; agul@mghihp.edu (A.G.); yarbel@mghihp.edu (Y.A.)

**Keywords:** developmental language disorder, feedback processing, probabilistic classification learning, encoding, event-related potential

## Abstract

Language acquisition depends on the ability to process and learn probabilistic information, often through the integration of performance feedback. Children with developmental language disorder (DLD) have demonstrated weaknesses in both probabilistic learning and feedback processing, but the individual effects of each skill are poorly understood in this population. This study examined school-aged children with DLD (*n* = 29) and age- and gender-matched children with typical development (TD; *n* = 44) on a visual probabilistic classification learning task presented with and without feedback. In the feedback-based version of the task, children received performance feedback on a trial-by-trial basis during the training phase of the task. In the feedback-free version, children responded after seeing the correct choice marked with a green border and were not presented with feedback. Children with TD achieved higher accuracy than children with DLD following feedback-based training, while the two groups achieved similar levels of accuracy following feedback-free training. Analyses of event-related potentials (ERPs) provided insight into stimulus encoding processes. The feedback-free task was dominated by a frontal slow wave (FSW) and a late parietal component (LPC) which were not different between the two groups. The feedback-based task was dominated by a parietal slow wave (PSW) and an LPC, both of which were found to be larger in the TD than in the DLD group. In combination, results suggest that engagement with feedback boosts learning in children with TD, but not in children with DLD. When the need to process feedback is eliminated, children with DLD demonstrate behavioral and neurophysiological responses similar to their peers with TD.

## 1. Introduction

Developmental language disorder (DLD) affects nearly 8% of English-speaking children [1,2]. It is a neurologically based impairment in using and understanding language that is not attributable to a genetic, biomedical, or acquired condition, nor to the acquisition of English as a second language [3]. The DLD label comprises a heterogenous group of children; however, most children with DLD experience difficulty learning words [4,5] and grammatical rules [6,7]. Learning is a complex skill that requires the processing of incoming information in a manner that facilitates storage and retrieval. How well new information is encoded may depend on the nature of the exposure to the input [8]. The learning mechanisms supporting language acquisition are poorly understood in children with DLD [9]. While researchers have made progress in identifying cognitive contributors to the learning challenges experienced by children with DLD, such as working memory [10,11,12,13], attention [14,15], and other executive functions [16,17,18], it is still unclear how different factors in the learning environment affect encoding in children with this disorder. One important factor that may affect how a learner interacts with the input—and ultimately influences how new information is encoded—is the presence of feedback during the learning process [19,20]. The present study evaluates the extent to which the presence of feedback affects stimulus processing and encoding of probabilistic information in children with DLD.

Feedback is a ubiquitous component of teaching [21], and it is often used by caregivers to shape the language development of young children [22,23,24]. Additionally, many clinical interventions rely on feedback to guide learning and shape performance. For example, interventions such as complex sentence treatment [25], shape coding [26,27], or focused stimulation [28] utilize feedback as part of structured and systematic cueing procedures. Some language interventions are designed to minimize the provision of feedback by withholding statements about accuracy and simply recasting or rephrasing a child’s incorrect productions [29,30,31]. This type of input is aimed at shaping performance while keeping the child unaware of errors, bypassing the need to interpret and use feedback to guide future actions. It is currently unclear which approach better supports learning in children with DLD. It is difficult to make equivalent comparisons between such intervention programs, given the complexities of the programs and the learning environments they produce. In addition to the presence or absence of feedback, intervention features such as the format of instructions, the content of treatment targets, and response modality are likely to affect performance and subsequent learning [32]. Because intervention is time and resource intensive, there is a need to isolate the effects of feedback within a controlled learning environment and determine which approach promotes better learning among children with DLD.

Feedback takes many forms, but one of its most basic functions is to provide input about the accuracy of a learner’s response [33]. Feedback processing differs depending on the learning environment and task demands [32,34]. In declarative learning paradigms, performance feedback is deterministic. This means that on a trial-by-trial basis, feedback provides consistent and unequivocal information about performance accuracy. Take, for example, a paired-associate learning task in which Word A is always paired with Object A. Skilled learners can usually determine the correct association by processing and utilizing feedback locally or after just a few trials. In probabilistic learning paradigms, performance feedback is less informative on a trial-by-trial basis. For example, on a complex category learning task, Objects A, B, and C might belong to Category 1 while Object D does not, despite sharing several features with the other objects. On this type of task, learners must accumulate information from feedback over time, resulting in more “global” processing. Learners ultimately acquire more general category classification rules than item-specific associations. 

Feedback-based learning has been reported to be atypical in children with DLD in tasks where feedback must be processed locally [19,20] or globally [35,36,37,38,39]. For example, work by Arbel and colleagues has confirmed that children with DLD achieve lower accuracy than their age- and gender-matched peers with typical development (TD) on both declarative [20] and probabilistic [35,36] learning paradigms. A trial-by-trial analysis of behavioral responses indicated that children with DLD were more responsive to positive feedback than negative feedback, regardless of the learning paradigm. In other words, they were more likely to repeat a correct response than to change it after positive feedback but were as likely to repeat an error as they were to correct it after receiving negative feedback. Electrophysiological data from both studies further supported a feedback-processing deficit among children with DLD. In particular, the feedback-related negativity (FRN) event-related potential (ERP) was absent during the declarative learning task [20] and significantly smaller in amplitude compared to peers with typical development during the probabilistic learning task [35]. Kemény & Lukács [37] also reported a deficit of feedback-based probabilistic learning among children with DLD, while Lee and Tomblin [38] observed this impairment among adults with a history of DLD. 

To fully understand the effects of feedback on learning outcomes, feedback must be isolated within the learning task. One way to achieve this is by examining performance on learning tasks presented both with and without feedback. For example, Arbel and colleagues [20] examined the effect of feedback on learning within a declarative task that required local, trial-by-trial processing. Specifically, they used a two-choice paired-associate word-learning task. Participants heard a spoken pseudoword and selected one of two novel objects. In the feedback-based version of this task, participants received information about the accuracy of their choice after each response, which requires continuous updating of memory for the correct associations. In the feedback-free version of this task, participants passively observed the correct pseudoword-object pairings for the same number of trials. All children, those with TD and DLD, learned more correct pairings in the feedback-free task than in the feedback-based task [20]. Feedback-free learning in this context can be viewed as “errorless” learning that reduces the reliance on working memory because the participants are only exposed to the correct associations. Given that working memory is a limited capacity resource, particularly for children with DLD [10,11,12,13], it is not surprising that children benefit from feedback-free declarative learning. These results are in line with previous reports of the potential advantages of errorless learning for typical children [40] and children with various neurodevelopmental disorders [41]. 

It is yet to be determined whether children with DLD benefit from a feedback-free learning environment when the task is probabilistic or requires global processing. Gabay and colleagues [42] examined the effect of feedback during probabilistic learning in young adults with and without developmental dyslexia—a language-based impairment of learning to read. They used the weather prediction (WP) task, which involves classifying sets of abstract cue cards as predictors of sunny or rainy weather based on their hidden statistical probabilities [43]. The typical WP task involves the use of performance feedback to promote learning through trial and error. The WP task can also be completed in a feedback-free condition, where cue cards and the correct outcome are presented simultaneously and learned by association. Results indicated that young adults with dyslexia achieved significantly lower accuracy than an age-matched control group in both the feedback-based and feedback-free learning conditions. The authors concluded that an impairment of probabilistic learning, rather than an impairment in feedback processing, accounts for the learning deficit in adults with dyslexia [42]. To the extent that children with dyslexia and DLD have similar language-based learning deficits [44], this study provides potential counterevidence to prior reports of feedback-processing deficits among children with DLD. It is also possible, however, that learning patterns in adults are different from those exhibited by children.

### The Present Study

This study was designed to evaluate input processing (i.e., stimulus and feedback processing) in children with DLD in a controlled learning environment that differs only by whether it is feedback-based or feedback-free. It is the first to examine whether children with DLD learn better with or without feedback when the learning task is probabilistic. This study is placed within an ongoing debate on whether the core learning deficit in DLD can be attributed to an impaired implicit learning mechanism (e.g., see [45]). Probabilistic learning is closely related to implicit learning, or the “acquisition of knowledge about the underlying structure of a complex stimulus environment by a process which takes place naturally, simply and without conscious operation” [46]. While probabilistic learning generally requires global feedback processing, the provision of feedback itself creates an environment that supports more local feedback processing and a shift toward explicit learning mechanisms [32]. Explicit learning is a conscious and intentional process used to form and store associations, facts, and rules [46,47]. Feedback-based probabilistic learning is likely to involve hypothesis testing, verification or rejection, and an increased reliance on memory on a trial-by-trial basis, all of which are considered explicit learning strategies. On the other hand, probabilistic learning without feedback is more likely to involve gradual and passive extraction of information over the course of several trials, which maintains the use of implicit learning processes [47]. To summarize, probabilistic learning in a feedback-based environment requires both implicit and explicit processing, while a feedback-free environment is more likely to draw upon gradual implicit processing. 

Using behavioral and electrophysiological measures, we seek to examine the effects of feedback on learning outcomes as well as the neural correlates associated with stimulus processing. Event-related potentials (ERPs) provide a non-invasive and temporally sensitive measure of cognitive processing that underlies behavioral responses. In other words, ERPs offer insight into the learning process that contributes to the behavioral learning outcome. Several studies have reported on ERP components associated with encoding [48,49,50]. Paller and Wagner [50] proposed that encoding relies on two processes: an initial process of transforming sensory information into internal mental representations and the second process of binding these representations into a persistent memory trace that enables subsequent recall [50,51].

The late parietal component (LPC), which is a centro-parietal positivity that peaks 400–700 ms post-stimulus [48,51,52], is suggested to be associated with the initial encoding process [50,53]. For example, in studies where participants were instructed to employ shallow strategies, such as rote repetition or subvocal rehearsal, they elicited a larger LPC amplitude for items later retrieved compared to items later forgotten [48,54]. An enhanced LPC effect was most prominent when individuals relied on rote strategies, weaker when they used elaborative strategies, and absent when they utilized more semantic-based strategies [48]. 

Slow waves are usually detected at both frontal and parietal electrodes approximately 800 ms post-stimulus [48,52,53,55] and are suggested to reflect hierarchically higher levels of cognitive processing of an event. The processing of a stimulus, according to the level of processing (LOP) framework, involves a hierarchy of cognitive functions ranging from early sensory processing to later in-depth analysis of conceptual features. In-depth or elaborative processing has been found to be associated with better encoding [56,57]. This distinction is also found to be reflected in ERP components that capture shallow/early sensory processing and those that reflect in-depth processing. More specifically, previous research has indicated that the LPC is related to encoding processes that are classified as “shallow”, while slow waves are related to encoding processes that are classified as “deep” [48,58]. The frontal slow wave (FSW) has been suggested to index associative encoding [52,59,60]. Its amplitude has been found to be sensitive to the strength of associations built through elaborative encoding processes [52,61]. The parietal slow wave (PSW) has been suggested to index the integration of components of an association into a unitized item representation [52,60]. For example, in a study by Kamp and colleagues [52], the PSW was elicited by word pairs that were presented with a definition that allowed participants to integrate the word pairs into a new concept. However, it was not detected when word pairs appeared with a sentence frame that did not support the creation of a new concept. Interestingly, the FSW was found in both conditions, strengthening the view that it reflects processes involved in encoding associations.

Given evidence of atypical feedback processing in DLD, we hypothesized that children with DLD would demonstrate better performance on the feedback-free version of a probabilistic learning task than the feedback-based version. These results would contrast with the implicit deficit account of DLD, as they would suggest that relying on implicit learning mechanisms is more beneficial for children with DLD. Given the results of Gabay and colleagues [42] with a similar population, we considered the possibility that children with DLD would demonstrate a deficit of probabilistic or implicit learning that is present regardless of the feedback environment when compared to age- and gender-matched peers with typical development. 

We also expected that stimulus processing would differ between the feedback-free and feedback-based tasks. To test this hypothesis, we compared patterns of activation for stimulus-locked ERPs between task and group. Both feedback-free and feedback-based probabilistic paradigms involve the learning of item-category associations across multiple trials. However, feedback-based learning places more cognitive demands on working memory, hypothesis testing, and decision-making than feedback-free learning, potentially resulting in more effective encoding [56,57]. Since the provision of feedback facilitates both the initial integration of sensory information and the later consolidation of mental representations into memory traces, children with typical development were predicted to better utilize feedback to support learning [50,51].

In the feedback-based task, where more explicit learning mechanisms are employed, we expected to find differences in the “shallow” processing between children in the two groups (TD, DLD) that will be reflected in reduced LPC amplitude in children with DLD, as well as differences in the deep processing reflected in the SW. The feedback-free paradigm was expected to be dominated by similar deep processing across groups reflected in similar SW activity.

## 2. Materials and Methods

### 2.1. Participants

Seventy-three children from the Boston area participated in this study. Participants were classified as having either developmental language disorder (DLD; *n* = 29) or typical language development (TD; *n* = 44) based on the criteria described below. All participants were right-handed individuals between the ages of 8 and 13 years (*M* = 10.29 years, *SD* = 1.39 years) with normal or corrected vision who reported no history of head injury or other neurological deficits and that English was their predominant language. All participants obtained a nonverbal intelligence score above the range of intellectual disability (SS > 80) on the Matrices subtest of the Kaufman Brief Intelligence Test, 2nd Edition (KBIT-2). 

Inclusion criteria for the DLD group were as follows: (a) Standard score below 85 on the Core Language Score, Receptive Language Index, or Expressive Language Index of the Clinical Evaluation of Language Fundamentals, 5th Edition (CELF-5) (Three participants met the criteria for DLD based on obtaining scaled scores of 7 or lower for at least 2 subtests of the CELF-5) or (b) Identification Core score on the Test of Integrated Language and Literacy Skills (TILLS) of less than 34 if 8–11 years old or less than 42 if 12–18 years old. Both the CELF-5 and TILLS are measures commonly used by speech-language pathologists to diagnose children with DLD. The CELF-5 has a reported sensitivity and specificity of 0.97 for scores that are −1.33 below the mean. The TILLS has a reported sensitivity range of 0.81–0.97 and a specificity of 0.81–1 for the age range of the participants in our study (8–13 years). All caregivers of children in the DLD group reported a history of delayed language development and persistent difficulties with spoken and/or written language. Children in the TD group obtained standard scores of 85 or above on all indices of the CELF-5 and no reported delays or difficulties with language. 

Table 1 presents inclusionary data by group as well as results of one-way analyses of variance (ANOVA) and chi-squared analysis. The DLD and TD groups did not significantly differ in age or proportion of males and females. There were significant group differences favoring those with TD on all other standardized assessments, as would be expected given the classification criteria. Participant caregivers also reported race and ethnicity on a digital questionnaire. In the TD group, 45% of participants identified as White, 18% identified as Asian, 11% identified as Black or African American, 11% identified as more than one race, and 14% identified differently from the provided options; 5% identified as Hispanic or Latino. In the DLD group, 62% of participants identified as White, 0% identified as Asian, 17% identified as Black or African American, 3% identified as more than one race, and 17% identified differently from the provided options; 21% identified as Hispanic or Latino.

### 2.2. Procedure

Participants were enrolled in a larger study on feedback processing containing 2–3 sessions lasting 90 min each. Standardized assessments were administered in 1–2 sessions before initiating the experimental tasks. The experimental tasks for the current study were administered on a computer while electroencephalography (EEG) was recorded from the scalp. Behavioral responses were collected using a Chronos response box. Each participant completed two classification learning tasks, lasting about 15 min each, in counterbalanced order. Each task contained a training phase, an immediate test, and a delayed test. The training and immediate test were completed during the research session, while the delayed test was completed approximately one week later from the child’s home computer. 

The data-collection and data-management procedures for this study were approved by the Mass General Brigham (MGB) Institutional Review Board. Informed consent and assent forms were provided to caregivers early in the recruitment process and resent a few days before the first lab visit. Caregivers had an opportunity to review the forms and discuss any questions or concerns with the research staff before signing. During the first lab visit, and before the initiation of data collection, verbal information about the study was shared with caregivers and children. Child-friendly language was used to describe the procedure to children. Children and caregivers were reminded that they could withdraw from the study at any point and were encouraged to alert the research staff of any discomfort. Data collection commenced after receiving signed and verbal consent from caregivers and children. Participants received a small prize at the end of each in-person lab visit and monetary compensation for completing the study.

### 2.3. Classification Learning Tasks 

The classification learning tasks were programmed using the E-Prime experiment generation software (E-Prime 2.0; Psychological Software Tools, Pittsburgh, PA, USA). The learning paradigm was based on work by Zeithamova and colleagues [65] and has been modified to be appropriate for children. A detailed description of the feedback-based task is reported in Gul et al. [35]. The goal of both tasks was to learn to correctly classify novel cartoon animals into one of two categories. The child-friendly framing of this goal involved deciding whether each animal belonged to the family that liked chocolate cupcakes or the family that liked vanilla cupcakes. Participants were reminded that, “family members look alike, but they don’t look exactly the same”. They were discouraged from focusing on a single feature of the stimulus and were asked to, “Go with your first guess, based on the overall impression”. All instructions were presented in writing while an audio recording read them aloud. The tasks were equivalent in terms of stimuli, overall training structure (i.e., four blocks with 40 trials each; 160 trials total), and test structure (i.e., immediate and delayed tests with 40 trials each). The tasks differed only in whether performance feedback was provided during training. 

#### 2.3.1. Stimuli

The animal stimuli differed in five binary features (e.g., round vs. square body shape, striped vs. spotted body pattern, etc.). The prototype for Category A differed from the prototype for Category B on all five features, while the exemplars contained a variable number of features from both categories (see Table 2). During training, participants were exposed to two types of exemplars (8 exemplars in total). Most exemplars (75% or six exemplars) differed from their prototype by one feature (i.e., they were one feature “away” from the prototype) and from the other prototype by four features. The remaining exemplars (25% or two exemplars) differed from their prototype by two features (i.e., they were two features away) and from the other prototype by three features. The prototypes were not presented during the training phase. The emphasis on training exemplars that shared features with the prototypes was intended to support the participant’s creation of a mental representation of each prototype. The probabilistic distribution of the features across different exemplars made it impossible to determine classification based on a single trial or any single feature. Participants had to gather knowledge over the course of many trials to learn the correct classification. Two sets of equivalent animal stimuli were counterbalanced across tasks and participants. During testing, participants saw the eight old exemplars (from the training), eight new exemplars, and the two prototypes. See Table 3 for the complete number of stimulus types and exposures.

#### 2.3.2. Feedback-Based Training 

**Figure 1:**
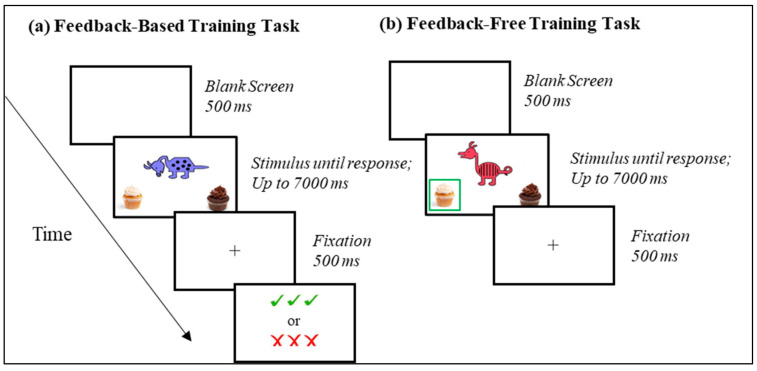
(**a**) Illustration of the trial structure in the feedback-based training task; (**b**) Illustration of the trial structure in the feedback-free training task.

During the feedback-based training, participants initially had to guess the category for each exemplar, then use feedback to validate or invalidate their choice and make adjustments for future responses. Each trial consisted of a blank screen for 500 ms, a stimulus screen for up to 7000 ms (or until a response was made), a fixation cross for 500 ms, and then a feedback screen for 1500 ms. The stimulus screen contained an exemplar in the center and the two category options (i.e., images of chocolate and vanilla cupcakes) in the bottom left and right corners. Participants were instructed to choose one of the two options by pressing the left- or right-most button on the response box. The feedback screen presented “√√√” following correct responses and “XXX” following incorrect responses. A feedback-based training trial is illustrated in Figure 1a. 

#### 2.3.3. Feedback-Free Training

During the feedback-free training, participants were instructed to observe the screen and press the button corresponding with the highlighted category. The button press encouraged participant engagement and advanced the task to the next trial. Each trial consisted of a fixation screen for 2000 ms and a stimulus screen for 7000 ms (or until a button press). The stimulus screen also presented an exemplar in the center with two category options in the bottom left and right corners; however, one of the options was outlined with a green box indicating the correct category classification for that exemplar. A feedback-based training trial is illustrated in Figure 1b. 

#### 2.3.4. Immediate Test

The test structure was identical for each task (feedback-based and feedback-free). An immediate test containing 40 trials followed each training phase (See Table 3). Participants were instructed to classify both old and new exemplars (including the prototypes) in the absence of feedback. They were asked to decide if an animal liked chocolate cupcakes or vanilla cupcakes and were instructed to “go with your first guess based on the overall impression”. Each test trial consisted of a fixation cross for 1000 ms followed by a stimulus screen for up to 7000 ms (or until a response was made). The stimulus screen contained an exemplar in the center and the two category options in the bottom left and right corners.

#### 2.3.5. Delayed Test

The delayed test was identical for each task, but it was modified to be administered remotely on the participant’s home computer. The same stimuli and response images from the immediate test were embedded in REDCap, a secure web platform for building and managing online databases and surveys. At the end of the in-lab research visit, participants completed several practice items (2 items from each experimental task) in a practice REDCap survey on the lab computer. One week after the research visit, the participant’s parent/guardian received an email invitation to open a similar REDCap survey and have their child complete the delayed test from their home computer.

### 2.4. Behavioral Data Analysis

Learning outcomes were obtained from each task to examine whether the effect of feedback on classification learning differed between children with DLD and TD. For each task, accuracy was examined on the immediate and delayed tests. Immediate test accuracy was further broken down by stimulus type. The stimulus type (i.e., prototypes, exemplars that are one feature away, and exemplars that are two features away) measures the impact of the stimuli’s statistical properties on learning. The accuracy for prototypes, or the ability to construct a prototype following exposure to exemplars, was also examined. 

Behavioral data were analyzed using a series of one-way and mixed analyses of variance (ANOVAs). For variables containing three or more levels, we examined the equality of variances using Mauchly’s test of sphericity. When indicated by a significant interaction effect, pairwise comparisons were analyzed with Bonferroni correction. All error bars represent the standard error of the mean. The prototypes and exemplars from each category (A and B) were designed to have the same statistical properties. This was confirmed via mixed ANOVA with group (TD, DLD) as a between-subject variable and category (A, B) as a within-subject variable for each task. For the feedback-based task, there were no significant differences in accuracy between categories A and B for prototypes, *F* (1, 68) = 0.74, *p* = 0.394, exemplars that were one feature away, *F* (1, 68) = 0.12, *p* = 0.730, and exemplars that were two features away, *F* (1, 68) = 0.43, *p* = 0.515. For the feedback-free task, there were no significant differences in accuracy between categories A and B for prototypes, *F* (1, 68) = 0.04, *p* = 0.839, exemplars that were one feature away, *F* (1, 68) = 1.32, *p* = 0.254, and exemplars that were two features away, *F* (1, 68) = 0.03, *p* = 0.864. Then, category values were averaged together, creating a single variable for each stimulus type that could be interpreted in the same way as the other accuracy variables. 

### 2.5. EEG Data Collection & Analysis

#### 2.5.1. EEG Data Acquisition 

EEG data were collected using the GES 400 System by Electrical Geodesics Inc. (EGI; Eugene, OR, USA) with 32-channel HydroCel Geodesic Sensor Nets from EGI. EEG was continuously recorded from all electrodes at a 1000-Hz sampling rate using the vertex as the reference electrode. The impedances of all electrodes were kept below 50 kΩ. All ERP data analyses described below were performed using custom-written MATLAB (The MathWorks, Inc.; Natick, MA, USA) scripts operating in conjunction with the open-source EEGLAB toolbox version 2021.0 [66]. All further ERP analyses described below were completed in MATLAB using the EEGLab toolbox and custom-written routines.

#### 2.5.2. EEG and Signal Processing

The EEG data were processed for each task separately. Continuous data were put through a bandpass filter (0.1–30 Hz) and were then segmented into 1200 ms epochs, using −200 ms before and 1000 ms after stimulus presentation (i.e., shortly before and after the animal exemplar was shown on the screen). Each trial was visually inspected and manually removed if artifacts were detected. Data were re-referenced to the average reference [67]. An Adaptive Mixture ICA (AMICA) was applied separately to single-subject datasets [68] to detect and correct for eye movement and eye blinks. Baseline correction was performed on the pre-processed clean data based on the signal in the 200 ms preceding the stimulus (i.e., −200 to 0 ms). 

#### 2.5.3. Event-Related Potential (ERP) Data Analysis 

ERP data analysis focused on the training phase in both tasks because we were interested in the processing of stimuli under the two learning conditions (i.e., feedback-based and feedback-free). Visual inspection of all electrodes revealed more differences at the fronto-central (FCz) and parietal electrodes (Pz). ERP data were extracted from these electrodes for each participant across all four training blocks using EEGLab’s statistical tools. To reduce the temporal dimensionality of the dataset for both electrodes, a temporal PCA (TPCA) with Promax rotation [69] was performed [70,71]. The covariance between time points is used in temporal PCA to disentangle ERP components that overlap in time. This analysis was performed separately on the data points from electrodes Pz and FCz, and the temporal factors obtained were statistically analyzed using IBM SPSS Statistics 24.0. (IBM, Armonk, NY, USA). Significant effects are reported with the Greenhouse–Geisser corrected degrees of freedom when appropriate.

## 3. Results

### 3.1. Behavioral Results

Table 4 summarizes test accuracy for each task and group. The following sub-sections examine immediate test accuracy, test accuracy by stimulus type, prototype learning, delayed test accuracy, and feedback-based training accuracy. 

#### 3.1.1. Immediate Test Accuracy

**Figure 2:**
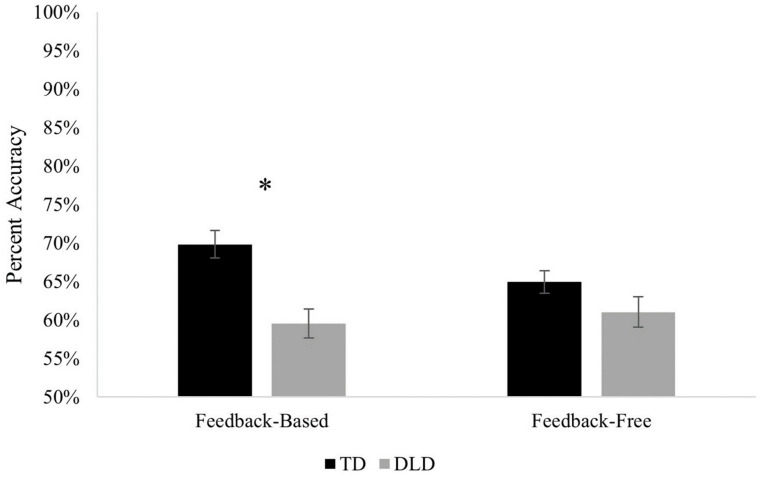
Overall test accuracy by task and group (* indicates *p* < 0.001).

Immediate accuracy during testing was evaluated using a mixed ANOVA with task (feedback-based, feedback-free) as a within-subject variable and group (DLD, TD) as a between-subject variable. The main effect of group was significant, *F* (1, 67) = 11.33, *p* = 0.001, *η_p_*^2^ = 0.145, with the TD group achieving higher accuracy than the DLD group. The main effect of task was not significant, *F* (1, 67) = 0.92, *p* = 0.340, *η_p_*^2^ = 0.014. However, a significant interaction between task and group was found, *F* (1, 67) = 4.08, *p* = 0.047, *η_p_*^2^ = 0.058. Post hoc analysis of the interaction indicated that the TD group performed significantly better than the DLD group on the feedback-based task (*p* < 0.001), but the groups did not significantly differ on the feedback-free task (*p* = 0.203) (Figure 2 illustrates this interaction). It was also the case that accuracy for the TD group differed on the two tasks (*p* = 0.022), but the DLD group performed similarly on both tasks (*p* = 0.494).

#### 3.1.2. Effect of Stimulus Type

The extent to which children extracted the probabilistic features of the stimuli was determined by examining test items as a function of distance from the prototypes. We conducted a mixed ANOVA with task (feedback-based, feedback-free) and stimulus type (prototype, one feature away, two features away) as within-subject variables and group (DLD, TD) as a between-subject variable. Mauchly’s test was statistically significant (χ^2^(2) = 8.74, *p* = 0.013); therefore, Greenhouse–Geisser corrected values are reported. A significant main effect of group (*F* (1, 67) = 10.42, *p* = 0.002, *η_p_*^2^ = 0.135) and significant interaction between task and group (*F* (1, 67) = 5.54, *p* = 0.021, *η_p_*^2^ = 0.076) replicated the findings for overall test accuracy. The main effect of task was not significant, *F* (1, 67) = 0.043, *p* = 0.836, *η_p_*^2^ = 0.001. The main effect of stimulus type was significant (*F* (1.78, 119.22) = 50.51, *p* < 0.001, *η_p_*^2^ = 0.430) with pairwise comparisons indicating that accuracy was significantly different for each stimulus type (all *p*-values < 0.001); it was greatest for the prototypes, then stimuli that were one feature away, and finally, stimuli that were two features away. The remaining interactions between stimulus type and group, stimulus type and task, and all three variables were not significant (all *p*-values > 0.070).

#### 3.1.3. Prototype Learning

**Figure 3:**
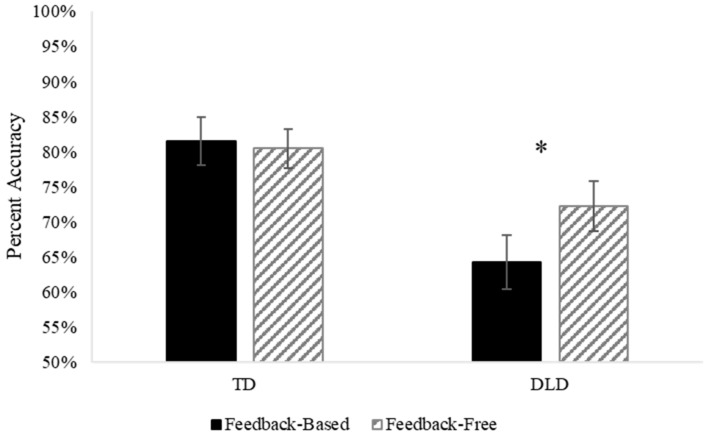
Prototype learning by group and task. An examination of prototype learning between the two conditions (with and without feedback) for each group (TD, DLD) separately, revealed a significant difference between conditions in the DLD group (*p* = 0.025, indicated with a *) but not the TD group (*p* = 0.715).

Because prototype learning represents a unique component of classification learning, we examined the accuracy for the prototypes separately from the other stimuli using a mixed ANOVA with task (feedback-based, feedback-free) as a within-subject variable and group (TD, DLD) as a between-subject variable. The main effect of group was significant, *F* (1, 67) = 8.70, *p* = 0.004, *η_p_*^2^ = 0.115, but the main effect of task was not, *F* (1, 67) = 2.35, *p* = 0.130, *η_p_*^2^ = 0.034. The interaction between task and group was not significant, *F* (1, 67) = 4.00, *p* = 0.050, *η_p_*^2^ = 0.056, but results indicated a trend in which the DLD group learned prototypes better in the feedback-free task (*M* accuracy = 0.72) than in the feedback-based task (*M* accuracy = 0.64). The TD group demonstrated similar prototype learning across tasks (*M* accuracy = 0.80 and 0.81, respectively). These results are illustrated in Figure 3. 

#### 3.1.4. Delayed Test Accuracy

Accuracy on the delayed test was evaluated using a mixed ANOVA with task (feedback-based, feedback-free) as a within-subject variable and group (DLD, TD) as a between-subject variable. No significant effects were observed for task, *F* (1, 57) = 0.28, *p* = 0.601, *η_p_*^2^ = 0.005, group, *F* (1, 57) = 0.01, *p* = 0.933, *η_p_*^2^ < 0.001, or the interaction between task and group, *F* (1, 57) = 0.90, *p* = 0.346, *η_p_*^2^ = 0.016. It is notable that only a sub-sample of participants completed the delayed tests with more data missing from the DLD group (*n* = 9) than the TD group (*n* = 3). Thus, we examined each group in a separate mixed ANOVA with time (immediate, delayed) and task (feedback-based, feedback-free) as within-subject variables. For the TD group, there was a significant main effect of time, *F* (1, 37) = 5.39, *p* = 0.026, *η_p_*^2^ = 0.127, and task, *F* (1, 37) = 4.44, *p* = 0.042, *η_p_*^2^ = 0.107, indicating that accuracy was better on the immediate than the delayed test and on the feedback-based condition than the feedback-free condition. For the DLD group, there were no significant differences in time, *F* (1, 18) = 0.22, *p* = 0.644, *η_p_*^2^ = 0.012, or task, *F* (1, 18) = 0.66, *p* = 0.426, *η_p_*^2^ = 0.035. The results for delayed test accuracy should be interpreted with caution, given that less than 75% of the DLD group completed this remote assessment compared to more than 90% of the TD group.

### 3.2. Event-Related Potential (ERP) Results

#### 3.2.1. Frontal Slow Wave (FSW) 

A temporal principal component analysis (TPCA) was conducted at the FCz electrode. The results of the TPCA analysis at electrode FCz indicated that six temporal factors accounted for 81.23% of the total variance. Of these factors, TF1, peaking around 800 ms, was identified as capturing the frontal slow wave (FSW). 

**Figure 4:**
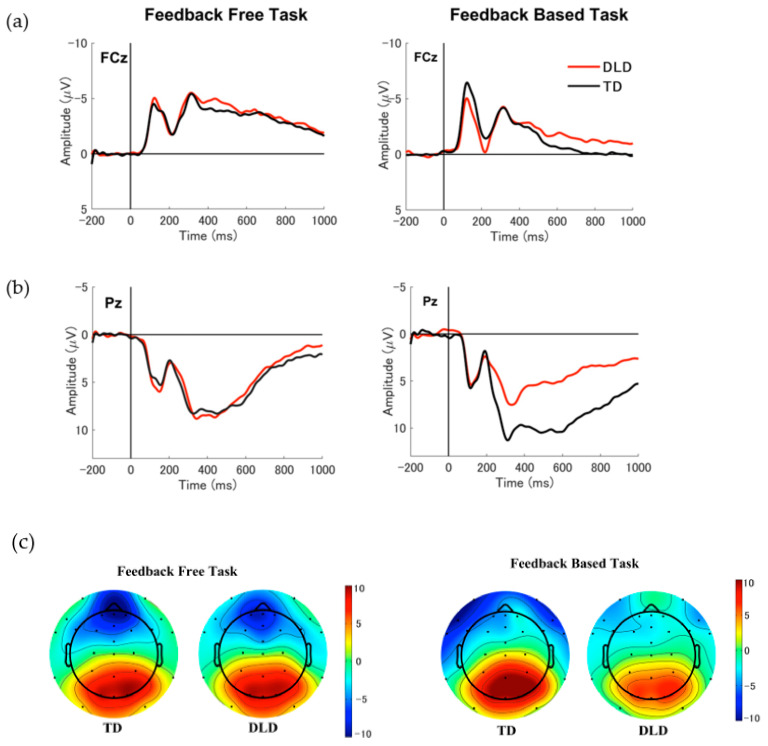
(**a**) Grand-average frontal slow wave (FSW) recorded at FCz for feedback-free and feedback-based tasks in children in TD (shown in black) and DLD (shown in red). (**b**) Grand-average parietal slow wave (PSW) and late parietal component (LPC) recorded at Pz for feedback-free and feedback-based tasks in children in TD and DLD. (**c**) Topographic maps are generated for both tasks, covering the time window of 450–600 ms.

**Figure 5:**
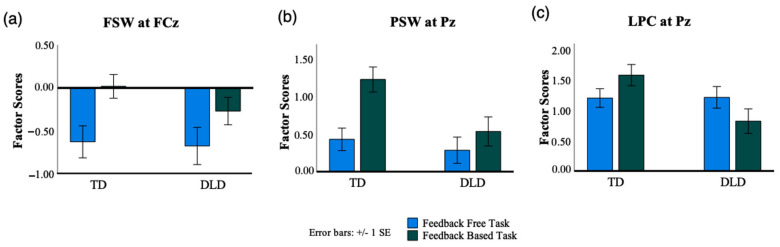
(**a**) Comparison of factor scores for frontal slow wave (FSW) at FCz between children with TD and DLD during the feedback-free task (shown in blue) and the feedback-based task (shown in green). (**b**) Comparison of factor scores for the parietal slow wave (PSW) at Pz between groups and tasks. (**c**) Comparison of factor scores for the late parietal component (LPC) at Pz between groups and tasks.

To examine the effect of probabilistic learning with and without feedback on the FSW in children with TD and DLD, a mixed ANOVA was conducted on TF1 factor scores. The analysis included task (feedback-free, feedback-based) as a within-subject variable and group (DLD, TD) as a between-subject variable. The results revealed a main effect of task, *F* (1, 59) = 16.166, *p* < 0.001, *η_p_*^2^ = 0.215, with the feedback-free task eliciting a larger FSW (more negative) than the feedback-based task across groups (see Figure 4a and Figure 5a). The main effect of group was not significant, *F* (1, 59) = 0.614, *p =* 0.436, *η_p_*^2^ = 0.010, and neither was the interaction between task and group, *F* (1, 59) = 0.838, *p* = 0.364, *η_p_*^2^ = 0.014. Overall, these results suggest that the FSW component was sensitive to task demands in both DLD and TD children, with the feedback-free task eliciting larger FSW than the feedback-based task.

#### 3.2.2. Parietal Slow Wave (PSW) and Late Parietal Component (LPC) 

A temporal principal component analysis (TPCA) was performed at the Pz electrode, yielding a set of eight temporal factors that accounted for 81.19% of the total variance. Temporal factor 1 (TF1) and temporal factor 2 (TF2) were identified by the TPCA analysis as capturing the PSW and LPC activation, respectively. 

**PSW.** To examine the effect of probabilistic learning with and without feedback on the PSW in children with TD and DLD, a mixed ANOVA was conducted with task (feedback-free, feedback-based) as a within-subject variable and group (TD, DLD) as a between-subject variable. The findings indicated significant main effects of group, *F* (1, 59) = 4.862, *p* = 0.031, *η_p_*^2^ = 0.078, and task, *F* (1, 59) = 11.801, *p* = 0.001, *η_p_*^2^ = 0.167. On average, PSW amplitudes were greater for children with TD than DLD and in the feedback-based task compared to the feedback-free task (see Figure 4b and Figure 5b). Despite this apparent trend, the analysis indicated no significant interaction between the task and group factors, *F* (1, 59) = 3.217, *p* = 0.078, *η_p_*^2^ = 0.052. 

**LPC.** A two-way mixed ANOVA with task (feedback-free, feedback-based) as the within-subject variable and group (TD, DLD) as the between-subject variable was employed. While no significant main effects of group, *F* (1, 59) = 2.121, *p* = 0.151, *η_p_*^2^ = 0.035, or task, *F* (1, 59) = 0.040, *p* = 0.843, *η_p_*^2^ = 0.001, were observed, a significant interaction between group and task was found, *F* (1, 59) = 12.184, *p* < 0.001, *η_p_*^2^ = 0.171) indicating that task affected LPC potentials differently for each group. To further explore the task and group interaction, a post hoc analysis was conducted. Results indicated that in the DLD group, the LPC amplitudes were significantly more positive in the feedback-free task compared to the feedback-based task (*p* = 0.005), while the pattern was reversed in the TD group (*p* = 0.028). The feedback-based task elicited significantly higher amplitudes in the TD group in comparison to the feedback-free task. Moreover, the LPC amplitudes in the feedback-based task were significantly higher in the TD group compared to the DLD group (*p* = 0.009), while no significant amplitude differences were found between groups in the feedback-free (*p* = 0.206).

### 3.3. Results Summary

Behavioral data analyses indicated that children in the TD group obtained greater accuracy than the DLD group on the feedback-based task; however, groups obtained similar levels of accuracy on the feedback-free task. For both groups, accuracy was greatest for the prototypes, then stimuli that were one feature away, and finally, stimuli that were two features away. When examining the prototypes alone, there was a clear, though not significant, pattern in which the TD group learned prototypes equally well across tasks, but the DLD group learned prototypes better in the absence of feedback compared to with it. 

ERP data analyses provided insight into the processing underlying behavioral performance, specifically, the encoding of stimuli in the feedback-based and feedback-free tasks. The feedback-free task was found to be dominated by a frontal slow wave (FSW), which was larger than that obtained in the feedback-based task but not different between the two groups (TD, DLD), and a late parietal component (LPC) that was not different between the two groups. The feedback-based task was dominated by a parietal slow wave (PSW) and an LPC, both of which were found to be larger in the TD than in the DLD group. In the DLD group, the LPC was found to be larger in the feedback-free than in the feedback-based task.

## 4. Discussion

The present study investigated the effect of feedback-based and feedback-free learning environments on encoding during a probabilistic classification learning task in school-aged children with and without DLD. The two versions of the task were nearly identical, with the presence or absence of feedback during training being the primary difference. The feedback-free training showed the correct response for each item simultaneously, while the feedback-based training indicated response accuracy following each response. At its core, a probabilistic classification task requires implicit learning to sort complex visual stimuli into the correct categories based on the gradual and unconscious learning of probabilistic feature combinations for each category. The introduction of performance feedback may encourage the learner to use more explicit strategies to intentionally create and test hypotheses on a trial-by-trial basis. In other words, probabilistic learning in a feedback-free environment is likely to follow the principles of implicit learning, while a feedback-based environment shifts resources toward feedback processing and a more explicit approach to learning. 

Our measures of encoding included behavioral learning outcomes as well as electrophysiological measures of stimulus processing during training. We predicted that children with DLD would demonstrate better learning in the feedback-free task than in the feedback-based task, suggesting impaired feedback processing. However, we also considered the possibility that children with DLD would perform poorly in both tasks due to an underlying deficit of implicit learning. We predicted that behavioral results across paradigms and groups would be underscored by patterns of ERPs that align with shallow encoding as measured by the late parietal component (LPC) and/or deep encoding as measured by the frontal and parietal slow waves.

Overall, the results supported the feedback-processing-deficit hypothesis. Children with TD achieved higher accuracy than children with DLD following feedback-based training, while the two groups achieved similar levels of accuracy following feedback-free training. Results also showed that children with TD demonstrated better learning when training was feedback-based than when it was feedback-free, but children with DLD performed the same in both learning environments. These findings are consistent with previous research from our lab [20,35,36] as well as with other studies of feedback-based probabilistic learning in children and young adults with DLD [37,38,39]. The combined evidence suggests that engagement with feedback boosts learning in typically developing children, while children with DLD have difficulty processing and using feedback to support learning. These findings contradict theories such as the procedural deficit hypothesis [45]) that credit implicit learning deficits for language learning difficulty. These theories would suggest that declarative learning mechanisms can compensate for and support implicit learning weaknesses in children with DLD. However, in the present study, the declarative mechanism, or the use of explicit feedback, did not support learning in children with DLD. Our interpretation that declarative learning strategies do not serve as a compensatory mechanism is in contrast with a previous report that explicit instructions enhanced the learning of novel grammatical forms in children with DLD [72] but in line with another report [73], where explicit instructions did not support statistical learning in children with this disorder. It is yet to be evaluated whether explicit support can be optimized to improve implicit learning in children with DLD. 

It is important to acknowledge that the type of feedback used in our paradigm was not informative on a trial-by-trial basis, which could have reduced the attention to and motivation for using feedback during learning [33]. Furthermore, the work of “translating” feedback from a statement about accuracy into a plan of action to achieve the learning goals may be more challenging for children with DLD due to their weaknesses in language, attention, and memory. This processing breakdown could occur at the point of interpreting feedback, recalling previous trials, integrating new feedback with a current mental representation, or making a prediction about a future trial. It is possible that children with DLD could improve performance on probabilistic classification tasks, regardless of feedback environment, given a greater amount of training, as was observed by Lee & Tomblin [38] among young adults with a history of developmental language impairments. Whether a greater number of exposures or a different type of feedback would be more beneficial is also a topic for future research.

Not all studies of children with language-based learning impairments have observed this feedback-processing deficit. In a study of young adults with dyslexia, Gabay and colleagues [42] found that probabilistic learning was impaired regardless of the feedback environment. This study used the weather prediction (WP) task with and without feedback. Like our task, the stimuli are probabilistic, but they are limited to a much smaller set of possible stimuli. Using four cards, a maximum of 15 card combinations can be presented in the task. Participants gradually learn which card combinations are associated with each of two weather outcomes. The same stimulus items from training are seen again during testing, but no novel items are presented given the constraints of the stimulus design. Therefore, it has been suggested that participants shift from probabilistic learning to declarative learning in the later stages of the WP task [34,37,43]. After repeated exposures, participants can use declarative memory to form more explicit associations between the stimuli and the correct classification. When the same stimuli are tested following a training period, the outcome being measured may be more representative of declarative memory for specific stimuli than the acquisition of a nondeclarative classification rule. Thus, it is possible that the discrepancy between the results of the present study and those reported by Gabay and colleagues [42] stems from paradigm differences that affect the dominance of the declarative and implicit learning mechanisms. The feedback-free probabilistic classification learning paradigm of the present study may be more consistently approximating implicit learning, which creates a more robust contrast with the feedback-based paradigm, which may encourage the involvement of declarative learning. 

The analysis of prototypes across groups and tasks was significant to this investigation because prototype accuracy represents the participants’ deep knowledge of the classification rule or their ability to detect the most probable member of each category [65]. Prototypes were not presented during training, but participants appeared to have constructed a mental representation of them as they were exposed to the training exemplars. This conclusion is based on the finding that, regardless of feedback condition, test accuracy decreased as distance from the prototypes increased. Accuracy was highest for prototypes in both tasks and across both groups. Notably, the TD group demonstrated similar levels of prototype learning across tasks, whereas the DLD group displayed superior prototype learning in the feedback-free task compared to the feedback-based task. The lack of a significant interaction to support this observation may be offset by different sample sizes and reduced power for the DLD group. If valid and reliable, this result would bolster our hypothesis that children with DLD exhibit better learning without feedback. Taken together, the results indicate that both groups were sensitive to the probabilistic nature of the stimuli, regardless of whether learning was feedback-based or feedback-free. 

The ERP results provide further insight into the neural processes underlying encoding in the two learning conditions. Patterns observed in the ERP data associated with stimuli processing in the two paradigms were aligned with the behavioral outcomes for each task. The feedback-free task was found to be dominated by a frontal slow wave (FSW), which was larger than that obtained in the feedback-based task but not different between the two groups (TD, DLD). These ERP results are in line with the behavioral findings that there are no performance differences between the two groups when they engaged in the feedback-free learning task. The frontal slow wave (FSW) has been suggested to index the strength of associative encoding [52,59,60] and has been consistently observed in situations where deep or elaborative encoding processes are crucial for later retrieval [52]. The observation of a frontal slow wave (FSW) during the feedback-free condition suggests that learners were engaged in associative encoding processes that support the formation of categories based on a probabilistic combination of features. The lack of processing differences between the two groups suggests that children with DLD may have engaged typical encoding processes when feedback was absent.

The late parietal component (LPC) in the feedback-free task was also not different between the two groups, consistent with the notion that the two groups engaged with the incoming stimuli in the same manner during the encoding process of the feedback-free task. The LPC has been linked to more shallow encoding processes, such as rote repetition or subvocal rehearsal [48,58], with larger LPC amplitudes found for items later retrieved compared to items later forgotten [48,54]. Interestingly, within the DLD group, the LPC was found to be larger in the feedback-free than in the feedback-based task, consistent with their behavioral performance patterns.

The feedback-based task was dominated by a parietal slow wave (PSW) and an LPC, both of which were found to be larger in the TD than in the DLD group. The LPC and a PSW-like component were observed in a previous study when several aspects of an experience were combined into a single-item representation [52], a process that could be seen as similar to that involved in the combination of features to create a category. The atypical processing captured by these ERP components in the DLD group may suggest that a learning environment that relies on the gradual extraction of information from feedback to combine probabilistic features into a prototype negatively affects the neural processing needed for effective encoding. Atypical feedback processing and feedback-based learning have been previously documented in behavioral studies of children with DLD [20,35,36,37,38,39]. Taken together, the behavioral and electrophysiological data of the present study suggest that classification learning without feedback is typical in children with DLD. The results also highlight the detrimental effect of a feedback-based learning environment on encoding during classification learning in children with DLD. 

### 4.1. Implications

These results have potential clinical and educational implications for children with DLD. Probabilistic classification learning, or the ability to sort stimuli into categories based on specific criteria, is an important part of natural language learning. This is a complex process that primarily occurs through implicit learning mechanisms, which many believe to be impaired among children with DLD [45,74,75]. The results of the present study indicate that while children with TD benefited from the provision of feedback to support their learning, children with DLD did not. These results, coupled with our previous findings of atypical feedback processing in children with DLD, may suggest that teaching approaches that rely on the need to process feedback are not optimal for children with DLD. To the extent that the processes required for probabilistic classification learning are shared with the processes of implicit language learning, language interventions should be designed to minimize the provision of performance feedback. Instead, clinicians, educators, and parents should focus on promoting exposure to correct forms of language, such as in recasting interventions [29,30,31]. This approach encourages implicit learning and gradually strengthens the child’s underlying knowledge and, ultimately, use of the correct language forms. 

In practice, it can be difficult to provide a truly feedback-free learning experience as children are astute observers of their environment who quickly become aware of task goals and their own task performance [32]. Two approaches that are largely considered to be “feedback-free” and effective for children with DLD are auditory bombardment [76] and recasting [29,30,31]. Bombardment involves short but intensive exposure to exemplars embedded in a natural context. There are generally no instructions, no responses required from the child, and no feedback provided. Recasting comes in many forms, but essentially involves restating a child’s spontaneous utterance in a corrected form, with at least some of the child’s original utterance intact. While there is no evaluation of the child’s statement as correct or incorrect, many children can infer the corrective nature of adult recasts [22,23,24]. When it can be achieved, the experience of “errorless learning” has been shown to have positive impact on motivation, encouraging children to persist and engage in difficult learning tasks [41].

### 4.2. Limitations

A relatively small sample size and variance in accuracy may have precluded the detection of a significant interaction between task and group for prototype learning. The observed trend of children with DLD to learn prototypes better during the feedback-free training is likely to strengthen with additional participants. A strength of the sample was that groups were well-matched on age and gender, ensuring that basic demographics did not overly influence results. 

For the delayed test, the DLD group had a particularly low response rate. We analyzed delayed test data separately for each group to minimize the effect of unequal group sizes and a systematically greater loss of data for the DLD group. Moreover, these results were not weighed heavily in our discussion of findings. It is unclear why children with DLD were less likely to complete the delayed test, but it is possible that their caregivers faced more obstacles in coordinating resources and obligations related to their child’s diagnosis [77].

## 5. Conclusions

This study examined the effect of feedback on encoding and learning processes in children with DLD. It provides behavioral and electrophysiological evidence that feedback provision does not support learning in children with DLD, a finding that corroborates other work from our lab investigating both declarative [19,20] and probabilistic [35,36] learning paradigms. Furthermore, the results suggest that the feedback-based learning environment impacted the process of encoding new information by children with DLD. The present study further demonstrates that probabilistic classification learning under feedback-free conditions (i.e., those that bypass the need to process performance feedback) is not different between children with and without DLD. This conclusion challenges the leading theoretical perspective that impaired implicit learning underlies the language difficulties observed in DLD. These findings shed light on the impaired cognitive skills that underly learning in DLD and can inform practice by shaping the role of feedback in language interventions. Future research should test this conclusion in more clinically relevant models of language learning and include measures of children’s spoken responses. There is also a need to determine what “dosage” of exposure to a feedback-free learning environment is optimal for maximizing language development. 

## Figures and Tables

**Table 1:** Group Comparison on Inclusionary Measures.

	DLD	TD	One-Way ANOVA Results		
Inclusionary Measures	*n* = 29	*n* = 44	*df*	*F*	*p*
Age (in months)	122.52 (18.47)	124.14 (15.59)	1, 71	0.16	0.688
KBIT-2 Matrices Score	98.34 (12.74)	114.14 (10.83)	1, 71	32.27	<0.001
CELF-5 Core Language Score	85.78 (8.66)	116.34 (14.87)	1, 69	94.15	<0.001
CELF-5 Expressive Language Index	88.87 (7.73)	123.48 (12.27)	1, 36	94.36	<0.001
CELF-5 Receptive Language Index	82.07 (10.73)	121.91 (10.81)	1, 35	121.79	<0.001
TILLS Identification Core Score ^a^	66.91 (15.01)	--	--	--	--
			**Chi-Squared Test** **Results**		
Sex:			***df***	***X*** **^2^**	***p***
− Female − Male	16 13	20 24	1, *n* = 73	0.66	0.416

Note. Values are presented as mean (standard deviation); Assessment data are standard scores. KBIT-2 = Kaufman Brief Intelligence Test, 2nd Edition [62]; CELF-5 = Clinical Evaluation of Language Fundamentals, 5th Edition [63]; TILLS = Test of Integrated Language and Literacy Skills [64]. ^a^ The TILLS was administered to 11 participants, 8 of whom did not meet criteria for DLD based on CELF-5 performance but whose parents reported a history of language delay or impairment.

**Table 2:** Example of Probabilistic Feature Distribution by Category and Stimulus Type.

Category	Stimulus Type	Feature 1	Feature 2	Feature 3	Feature 4	Feature 5
A	Prototype	1	1	1	1	1
A	1	0	1	1	1	1
A	2	0	0	1	1	1
B	Prototype	0	0	0	0	0
B	1	1	0	0	0	0
B	2	1	1	0	0	0

Note. The numbers 1 and 0 reflect the two options for each feature presentation. For example, if Feature 1 is body pattern, 1 indicates stripes and 0 indicates spots. Prototype A differs from Prototype B on all five features while stimulus types 1 and 2 differ on one to four features from either prototype.

**Table 3:** Number of Stimuli and Exposures by Type.

	Category A Stimuli			Category B Stimuli				
	Prototype A	Exemplars 1 Feature Away from A	Exemplars 2 Features Away from A	Exemplars 2 Features Away from B	Exemplars 1 Feature Away from B	Prototype B	Total Number of Items	Total Number of Trials
Training Phase	0	3	1	1	3	0	8	160 training trials 20 × each exemplar
Testing Phase								
− Old Items	0	3	1	1	3	0	8	40 testing trials 2 × each exemplar 4 × each prototype
− New Items	1	2	2	2	2	1	10	

Note. The number of stimuli and exposures was the same for each task condition and test (feedback-based and feedback-free).

**Table 4:** Percent Accuracy by Task and Group.

	Feedback-Based		Feedback-Free	
	DLD (*n* = 28)	TD (*n* = 42)	DLD (*n* = 29)	TD (*n* = 41)
Overall Test Accuracy				
Immediate	60 (8)	70 (12)	61 (11)	65 (12)
Delayed ^a^	63 (10)	65 (10)	64 (11)	62 (9)
Breakdown of Immediate Test Accuracy by Stimulus Type				
Prototype	64 (21)	81 (22)	72 (19)	80 (18)
1 Feature Away	62 (13)	71 (14)	62 (14)	64 (15)
2 Features Away	54 (15)	60 (14)	55 (14)	56 (15)

Note. Values are presented as mean (standard deviation). ^a^ Sample sizes differed for the feedback-based follow-up test (DLD *n* = 21; TD *n* = 39) and the feedback-free follow-up test (DLD *n* = 20; TD *n* = 39).

## Data Availability

The data presented in the current study are available from the corresponding author upon reasonable request.
